# South West Urologists' Club, May 1990 Meeting

**Published:** 1991-03

**Authors:** 


### South West Urologists Club

### Meeting in Bristol, May 1990

### LONGTERM FOLLOW UP OF RENAL CARCINOMA WITH VENOUS INVOLVEMENT

**D. A. Gillatt, R. Persad, A. Weale, M. Horrocks, P. J. Smith**
Departments of Urology and Vascular Surgery, Bristol Royal Infirmary

Carcinoma of the kidney accounts for 1–2% of all malignancies. Surgery remains the only 'curative therapy available with the prognosis worsening with advancing stage. Renal cell carcinoma is unique in producing tumour thrombi which grow along the renal vein, inferior vena cava and may ultimately invade the right atrium. Although such venous involvement places the tumour in a higher stage (stage 3) several authors have suggested that radical excision of the primary lesion with the thrombus may be associated with a good prognosis. It has also been shown that the presence of lymph node involvement conveys a worse prognosis than venous involvement alone.

Eighty-two patients undergoing surgery for renal cell carcinoma at the Bristol Royal Infirmary between 1983 and 1988 have been studied. In 34 cases the tumour had breached the renal capsule and in 47 cases it had not. Fifty-eight cases showed no sign of venous involvement on pathological review whereas 26 patients had venous involvement and of these 9 had tumour within the inferior vena cava. The 3-year survival of the group was 63% for patients witout venous involvement 19% for those with venous involvement (both renal and inferior caval). Despite the apparently poor prognosis for venous involvement it is notable that some patients have had a good outcome. For example 6 out of 9 patients with caval involvement are still alive with a mean survival of 28 months (range 6–60 months). These are the patients who have been meticulously staged and selected for surgery. The patients who died early after caval exploration died from 1) lung metastases which were almost certainly missed at the time stagingand 2) postoperative cardiorespiratory problems. In conclusion we think that more radical surgical approach to patients with venous involvement is justified and is likely to improve survival significantly. However patients with caval involvement should undergo meticulous staging to rule out the presence of distant metastases and to accurately define the extent of venous involvement. They should also be medically fit in order to withstand such major surgery.

### IMAGING THE PROSTATE

**D. J. Chadwick**

Imaging the prostate is still a relatively new concept. There are however a number of reasons why imaging the gland may improve the management of prostatic disease. These include the early detection and accurate staging of cancer and volume measurement for monitoring the medical management of benign prostatic hyperplasia.

Methods used to image the prostate include computerised tomography (CT), transrectal ultrasound (TRUS) and magnetic resonance imaging (MRI). CT scanning has not been found to be useful in clinical practice due to its lack of tissue differentiation and the presence of artifacts from bone which occur during pelvic imaging.

TRUS is the most sensitive imaging technique. It has a role in screening for prostate cancer being able to visualise tumours as small as 4 mm in diameter. It has the advantage of allowing accurate guided biopsy via the transrectal or transperineal route. When used in staging patients with carcinoma of prostate TRUS is accurate in the detection of capsular breach as well as providing some information on the status of the seminal vesicles. In the assessment of the benign prostate TRUS allows accurate volume estimation using a planimetric method. Preoperative measurement of the length of the prostatic urethra is useful in the selection of a prostatic stent. In the investigation of inflammatory disease of the prostate TRUS detects prostatic abscess and allows guided needle aspiration when indicated.

MRI is a more expensive and time-consuming imaging modality. In the staging of patients with carcinoma of prostate MRI complements TRUS by providing further information regarding invasion of the seminal vesicles whilst in addition allowing imaging of the bladder base, pelvic lymph nodes and the bones of the pelvis and spine. The ability to create primary images in three planes and the excellent tissue differentiation obtained contribute to its superiority over CT. The recent development of endorectal coils has allowed greater resolution of the MR image due to the increase in signal to noise ratio.

In summary, imaging the prostate has improved the management of prostatic disease. TRUS remains the technique of choice for the diagnosis of prostate cancer and for the assessment of the benign gland. TRUS in combination with MRI permits accurate staging of malignant disease and allows accurate patient selection for curative or palliative therapy.

### HYDROSTATIC DILATATION OF THE URETER FOR URETEROSCOPY

**G. N. A. Sibley, M. I. Wills, V. K. George and S. C. W. Harrison**
Bristol Royal Infirmary and Southmead Hospital, Bristol

A new technique for hydrostatic dilatation of the ureter is described which is both simple and cost effective.

To gain access to the ureter, the intramural ureter is distended using a high pressure stream of the irrigant solution. The pressure is generated using a standard inflatable pressure infusor of the type more usually used for the rapid infusion of intravenous fluid. After traversing the intramural ureter, the irrigation is switched to a standard gravity fed system. This technique was assessed in 50 patients, including 1 patient undergoing bilateral ureteroscopy (39 ureteric stones, 8 diagnostic explorations and 4 ureteric stent retrievals).

This technique of dilation allowed successful introduction of the ureteroscope in 94% of cases. The subsequent ureteroscopic procedure was successfully achieved in 84% of cases. A failed outcome to ureteroscopy was usually due to inability to negotiate a tortuous ureter, although 2 stones were flushed back to the kidney during instrumentation (and then treated by ESWL). Complications included a ureteric performation in one case, and transient post-operative pyrexia in 11%, despite prophylactic antibiotic cover. Follow-up studies by IVP and DMSA renography have confirmed satisfactory results, with good preservation of renal function.

In conclusion, hydrostatic dilatation of the ureter has proved to be a valuable aid to successful ureteroscopy. The technique described uses existing, readily available theatre equipment and avoids the need for purchasing additional expensive apparatus.

### LONGTERM RESULTS OF TREATMENT WITH INTRAVESICAL BCG FOR RECURRENT SUPERFICIAL BLADDER CANCER

**A. Mukherjee, R. Persad and P. J. B. Smith**
Dept. of Urology, Bristol Royal Infirmary

Intravesical BCG has been used as a therapeutic and prophylactic agent in the management of superficial transitional cell carcinoma of the bladder. In the present study we wished to assess the *longerm efficacy* of intravesical BCG in the *prophylaxis* of superficial bladder carcinoma and to compare the efficacies of two well known strains of the vaccine (Pasteur and Glaxo). The latter to test the hypothesis that the Pasteur strain is a more effective agent than the Glaxo strain in prophylaxis. Twenty-one patients with recurrent superficial carcinoma of the bladder were randomised to receive either a) Glaxo (n = 12) or b) Pasteur (n = 9) strain of BCG. The vaccine was instilled diluted in 100 mls of normal saline (the dose of BCG used containing $1.2 \times 10^9$ colony forming units). The retention time in the bladder was one hour and treatment was given within 10 days of fulguration of the tumour. Further treatments were given at monthly intervals depending on the initial response as assessed at check cystoscopy. Patients were judged to be complete responders if there was endoscopic absence of tumour together with negative biopsies and negative urinary cytology. Lack of response or disease progression were judged as failures. Those patients in whom the tumour recurrence rate was reduced by at least 50% following treatment with the vaccine compared to before the vaccine had been given were judged to be 'partial responders'.

*Results:* At one year follow-up there was a 55% complete response rate for the Glaxo group and a 58% response rate for the Pasteur group. Of the nine failures at one year two patients had bladders clear of tumour but one had developed metastatic disease and the other had developed invasive urethral disease. Complications were minimal with transient haematuria and dysuria in 70% of cases only. At 60 months mean follow-up the complete response rate for the Glaxo groupo was 44% and for the Pasteur group was 41%. Of the 12 failures at 60 months mean follow-up (range 38–72 months) there were actually 4 (19%) patients who could be categorised as partial responders to the treatment.

*Conclusion:* The results of a 42% complete tumour-free rate and a 19% partial response at 60 months in this study compare well with other series (Morales *et al*, 1984, had 48% tumour-free rate at 60 months follow-up with a partial response of 17%). We were also unable to show any significant difference in the efficacy of the Glaxo strain as compared to the Pasteur strain of the vaccine for tumour prophylaxis. It would seem from these results that intravesical BCG is a worthwhile option in the prophylactic treatment of patients with multiple recurrent superficial transitional cell tumours requiring frequent cystoscopic fulguration. BCG is locally effective only conveying no systemic antitumour effect. We found no significant therapeutic side-effects or complications.

### MAGNETIC RESONANCE IMAGING FOR STAGING BLADDER CANCER

**R. Persad, D. Gillatt, J. Penry, J. Gingell, P. Smith**
Departments of Urology, Southmead Hospital and Bristol Royal Infirmary, Bristol

Accurate staging of transitional cell carcinoma of the bladder is essential prior to treatment. 24% of patients with extravesical spread have metastases compared to 15% of patients with disease confined to the muscularis and less than 1% where the disease is confined to the mucosa.

Conventional clinical staging may be inaccurate in 20–40% of cases. Computed Tomographic (CT) staging accuracy ranges between 50% and 70% in different series but some workers have demonstrated up to 80% false positive and 90% false negative results for the identification of involved lymph nodes.

Magnetic resonance imaging (MRI) was used to stage 55 patients with bladder carcinoma. T1 weighted spin echo (TR 500 ms, TE 26 ms), multi-echo (TE 1800 ms and TR 80 & 30 ms) and STIR (short tau inversion recovery) sequences (TR 1500 ms, TI 100 ms) were employed using a Picker Vista 2055 HP 0.5 Tesla scanner. Surgical correlation was obtained by transurethral resection and bimanual examination in 30 patients. 25 patients underwent cytectomy, laparotomy or post-mortem allowing more accurate correlation.

*Results*: The bladder tumours were well outlined by urine internally on all sequences and showed excellent contrast with surrounding fat on the T1 weighted sequence in particular.

The tumours appear of medium to low signal on T1 weighted sequences medium to high on T2 weighted and proton density sequences and high signal on STIR sequences. Involved lymph nodes show similar signal characteristics.

All Ta, T1 and T2 tumours were staged appropriately as being confined to the bladder wall. Of the 25 patients who had open surgery or post-mortem correlation with the MRI findings 19(76%) were accurately staged. Considering only the invasive tumours 17 out of 23 (73%) patients were accurately staged.

Two T3b tumours were overstaged as showing invasion of local organs. Two T3b tumours were understaged with regard to lymph node status only (regional lymph nodes removed at surgery were found to have micrometastases). Two T4 tumours were understaged, one with involvement of the prostate and the other with invasion at the bladder base. One of these patients also had undetected lymph node metastases. As regards lymph node staging there were only 3 false negative and no false positive findings out of twenty-four cases examined.

In conclusion, MRI compares favourably with other imaging techniques for the staging of bladder carcinoma producing clearer iamges due to its tissue specificity and also the ability to scan the pelvis in 3 planes. In common with CT micrometastatic involvement of lymph nodes may be overlooked (although the detection of involved lymph nodes is probably better with MRI than with CT) and invasion of the bladder base and prostate are areas of potential difficulty.

### TRANSCERVICAL ENDOMETERIAL RESECTION

**James Browning Nuala Dwyer**

Bristol

Hysterectomy for dysfunctional uterine bleeding is performed 40,000 times yearly in the UK. It is costly and has considerable physical and psychological morbidity. Selective endometrial ablation has the advantage of being a day-case treatment, whereby the patient may return to work the following day. Fibreoptic hysteroscopy may be harnessed with a standard urological resectoscope and glycine irrigation to remove the endometrium under direct vision. Diathermy resection to a depth of some 4 mm is adequate. Glycine deficit is carefully monitored.

The Bristol experience of this technique with early follow-up in 60 patients is discussed, and an exemplary video shown.

The early complications are of perforation, haemorrhage and fluid overload; haematometra with cervical stenosis and persistent menorrhagia may occur as late events. This is a new technique which we believe needs proper evaluation: to this end a prospective randomised trial against abdominal hysterectomy is being performed in Bristol.

### Wessex Branch of the Association of Clinical Pathologists: Winter Meeting

### Held at the Postgraduate Centre of the Royal Devon & Exeter Hospital, Thursday 6 December 1990

### THE DIFFERENTIAL DIAGNOSIS OF LYMPHOMAS AND GERM CELL TUMOURS USING IMMUNOHISTOCHEMISTRY

**M. Sweerts, P. J. Hall, N. Rooney**

Bristol Royal Infirmary

